# Therapeutic Mechanism of Lapatinib Combined with Sulforaphane on Gastric Cancer

**DOI:** 10.1155/2021/9933274

**Published:** 2021-09-18

**Authors:** Huixing Yi, Zheming Li, Xiaoxi Liu, Shijie Dai, Shouye Li

**Affiliations:** ^1^Department of Emergency, First Affiliated Hospital of Gannan Medical College, Ganzhou, Jiangxi 341000, China; ^2^College of Pharmacy, Hangzhou Medical College, Hangzhou, Zhejiang 310000, China; ^3^Medical College, Shaanxi Institute of International Trade and Commerce, Xianyang, Shaanxi 712046, China

## Abstract

**Background:**

Lapatinib is a small-molecule tyrosine kinase inhibitor that plays important roles in cell proliferation and survival. Administration of lapatinib with capecitabine is an effective treatment for HER2-positive metastatic BC. However, the effects of lapatinib on gastric cancer (GC) remain to be clear. In this study, we aimed to investigate the therapeutic effects of lapatinib combined with sulforaphane on GC and its underlying mechanisms.

**Methods:**

SGC-7901 and lapatinib-resistant SGC-7901 cells were treated with lapatinib (0.2 *μ*M), sulforaphane (5 *μ*M), or their combinations. Cell viability, invasion, cycle, and apoptosis of SGC-7901 and lapatinib-resistant SGC-7901 cells were evaluated by thiazolyl blue tetrazolium bromide (MTT), Boyden chamber assay, and flow cytometer. The protein expressions of HER-2, p-HER-2, AKT, p-AKT, ERK, and p-ERK were detected by Western blotting.

**Results:**

We observed that lapatinib combined with sulforaphane significantly decreased cell viability and inhibited cell migration of drug-sensitive and drug-resistant cells. Lapatinib sulforaphane also remarkably induced cell apoptosis with G0/G1 arrest. In addition, Western blotting revealed that the expressions of HER-2, p-HER-2, AKT, p-AKT, ERK, and p-ERK were downregulated by lapatinib-sulforaphane treatment.

**Conclusion:**

Combination of lapatinib and sulforaphane might be a novel and promising therapeutic treatment for lapatinib-sensitive or lapatinib-resistant GC patients.

## 1. Introduction

Gastric cancer (GC) is one of the most common malignant tumors worldwide, with high morbidity and mortality [1]. In 2018, there were 1,033,701 new cases of GC and 782,685 GC related deaths all over the world [1]. Moreover, 60% of world total cases and the highest incidence rates occur in Eastern Asia [1]. Although the diagnosis and treatment methods of GC have greatly improved, the five-year survival rate of GC patient is still 10–30% [2]. Development and progression of GC are regulated by many environmental and genetic risk factors, including *Helicobacter pylori* (*H. pylori*) infection, unhealthy dietary habits, alcohol consumption, smoking, obesity, gastric diseases, and others [3]. The routine treatments of GC include surgery, chemotherapy, and radiotherapy. Recently, molecular targeted treatment for GC has gradually deepened, which has greatly improved the efficiency of GC treatment and played an important role in the individualized treatment of GC [4, 5].

### 1.1. Up To

Lapatinib is the first dual inhibitor of epidermal growth factor receptor (EGFR) and human epidermal growth factor receptor 2 (HER2) tyrosine kinases, which reversibly binds to the cytoplasmic ATP-binding site of the kinase domain and blocks signal transduction cascade from the receptor [6]. This interaction leads to decreased growth, metastasis and angiogenesis, increased apoptosis, and genetic instability by affecting the Ras/Raf mitogen-activated protein kinase (MAPK) and phosphatidylinositol 3 kinase (PI3-K)/AKT signaling pathway [7]. Recently, increasing evidence indicated that HER2 might play an important role in the development and progression of GC [8]. Several preclinical studies have reported the antitumor activity of lapatinib in HER2-positive GC cell lines, suggesting the therapeutic effects of lapatinib for GC [9]. In a randomized placebo-controlled phase II study, lapatinib with epirubicin, cisplatin, and 5-fluorouracil or capecitabine was effective in GC patients, but not to patients with metastatic GC [10]. In addition, primary or acquired resistance to lapatinib still occurs in cancer therapy [11].

Sulforaphane is a natural isothiocyanate derived from cruciferous plants, such as broccoli, brussels sprouts, and cabbage. Sulforaphane has antioxidant and anticancer properties by triggering cell cycle arrest, apoptosis, and angiogenesis prevention [12]. In pancreatic cancer cells, sulforaphane inhibits sonic hedgehog (SHH) and PI3/AKT signaling pathway by reducing NF-kB's DNA binding capacity [13]. Furthermore, Mondal et al. reported that sulforaphane induced apoptosis and inhibited migration of human GC cells [14]. In vivo, studies presented that sulforaphane suppressed azoxymethane-induced colonic aberrant crypt foci (ACF) and prevented polyps in Apc/Min mice [15, 16]. Importantly, sulforaphane combined with other anticancer compounds could be more effective to target multiple pathways involved in cancer. For example, sulforaphane enhanced the efficacy of imatinib and sorafenib in pancreatic cancer cells and chronic myeloid leukemia cells, individually [17]. In HER2-positive breast cancer (BC) cells, sulforaphane not only potentiated antiproliferative activity of lapatinib but also was related to more effective induction of apoptosis [18]. However, the efficacy of combination therapy involving lapatinib and sulforaphane in GC remains unknown.

Therefore, we hypothesized that lapatinib combined with sulforaphane might be more effective in the treatment of GC. In this study, we investigated the molecular mechanisms associated with the synergistic antitumor effects observed following treatment with lapatinib and sulforaphane, as well as the efficacy of interactions between lapatinib and sulforaphane in SGC-7901 and lapatinib-resistant SGC-7901 cells.

## 2. Materials and Methods

### 2.1. Cell Culture and Reagents

GC cell lines SGC-7901 was obtained from the cell repository of Chinese Academy of Sciences (Shanghai, China) and cultured in RPMI 1640 medium supplemented with 10% fetal bovine serum, 100 unit/mL penicillin, and 100 *μ*g/mL streptomycin at 37°C in a 5% CO_2_ incubator. Lapatinib-resistant SGC-7901 cells were developed by 24 h exposure of SGC-7901 cells to 1 *μ*M concentrations of lapatinib. The primary antibodies against HER2, p-HER2, AKT, p-AKT, ERK, and p-ERK were bought from Cell Signaling Technology (Danvers, MA, United States). DMSO, thiazolyl blue tetrazolium bromide (MTT), the anti-*β*-actin, anti-mouse, and anti-rabbit antibodies were purchased from Sigma (St. Louis, MO, USA). The antibodies against HER-2, p-HER-2, AKT, p-AKT, ERK, p-ERK, and *β*-actin were from Cell Signaling Technology (Danvers, MA, USA) and Santa Cruz Biotechnology (Santa Cruz, CA, USA).

### 2.2. Cell Viability Assay

SGC-7901 and lapatinib-resistant SGC-7901 cells were mixed in different proportions (percentage of lapatinib-resistant SGC-7901 cells was 0, 5, 10, 25, 50, 75, or 100%). Combination cells (1 × 10^6^/mL) were seeded into 96-well culture plate and incubated at 37°C and 5% CO_2_ for 24 h. Then, the medium was removed, and cells were treated with lapatinib (0.2 *μ*M), sulforaphane (5 *μ*M), or combinations of compounds. After 48 h, 20 *μ*L MTT (5 mg/mL) was added to each well and cultured for 4 h. DMSO (200 *μ*L, Sigma, USA) was added and absorbance was measured at 570 nm using microplate reader (Bio-Rad, CA, USA).

### 2.3. Invasion Assay

Migration in vitro was assayed using BD BioCoat™ MatrigelTM Invasion Chamber. SGC-7901 and lapatinib-resistant SGC-7901 cells (1 × 10^5^ cells/well) were seeded in serum-free medium supplemented with the tested agents and the inserts were pre-coated with Matrigel (BD, USA). After cells were incubated for 24 h at 37°C, nonmigrating cells in the upper chamber were removed. The migrated cells were fixed with 4% formaldehyde solution for 10 min and then stained of 0.4% crystal violet in 10% ethanol for 5 min. The number of migrated cells was counted under the microscope (Olympus, Japan).

### 2.4. Cell Cycle Assay

SGC-7901 and lapatinib-resistant SGC-7901 cells (5 × 10^5^/well) were cultured in 6-well plates and incubated with lapatinib (0.2 *μ*M), sulforaphane (5 *μ*M), or combinations of compounds for 24 h. The cells were trypsinized and rinsed twice with cold PBS and resuspended in 70% cold ethanol at 4°C overnight. Then, cells were washed twice with cold PBS, incubated with 10 *μ*g/ml RNAse at 37°C for 30 min, and stained with Propidium iodide (PI) solution in the dark for 5 min. Finally, samples were analyzed by FACSCalibur flow cytometer (BD, USA).

### 2.5. Apoptosis Assay

Annexin V-FITC/PI apoptosis detection kit (Nanjing Jiancheng, China) was used to measure the apoptosis of SGC-7901 and lapatinib-resistant SGC-7901 cells in a similar procedure as above but without fixing in 70% ethanol. The cells were washed with PBS and resuspended in 1x binding buffer. Next, Annexin V-FITC and PI were added to stain for 15 min. The apoptosis cells were measured using the flow cytometer (BD, USA).

### 2.6. Western Blotting

SGC-7901 and lapatinib-resistant SGC-7901 cells were seeded in 4 ml of medium in 6 cm plate for 24 h, and the medium was replaced with lapatinib (0.2 *μ*M), sulforaphane (5 *μ*M), or combinations of compounds. After 48 h, total proteins were extracted with RIPA lysis buffer (Beyotime Biotechnology, China). The samples were loaded and separated by 12% sodium dodecyl sulfate-polyacrylamide gel electrophoresis and then transferred onto nitrocellulose membranes. The membranes were washed with PBS and blocked in 5% skim milk, followed by immunoblotted using the following primary antibodies: HER-2, p-HER-2, AKT, p-AKT, ERK, p-ERK, and *β*-actin overnight at 4°C. The anti-mouse and anti-rabbit antibodies conjugated with HRP were used for incubation. The proteins were visualized using the enhanced chemiluminescence (ECL) kit. Finally, the intensity of Western blot bands was analyzed with Quantity One 1D Analysis Software (Bio-Rad, USA).

### 2.7. Statistical Analysis

The results were presented as the mean ± standard deviation and analyzed by SPSS 16.0 software (IBM, USA). We performed the graphics using GraphPad Prism software (USA). Differences between groups in invasion tests were analyzed with Student's *t* test. *P* < 0.05 was considered as significantly different.

## 3. Results

### 3.1. The Effects of Lapatinib, Sulforaphane, and Their Combination on Viability of Drug-Sensitive and Drug-Resistant SGC-7901 Cells

**Figure 1:**
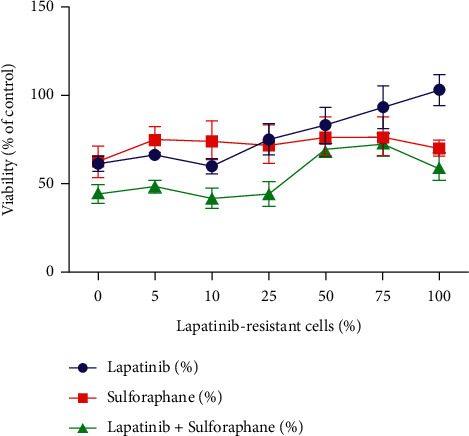
The effect of lapatinib, sulforaphane, and their combination on cell viability of SGC-7901 and lapatinib-resistant SGC-7901 cells were measured by MTT assay. Each point is mean (±SE) of two experiments done in triplicate.

To determine the influence of combined treatment on viability of the two cell lines, we performed the MTT assay. According to previous studies, we treated SGC-7901 and lapatinib-resistant SGC-7901 with 0.2 *μ*M sulforaphane, 5 *μ*M lapatinib, or their combinations for 48 h. As shown in Figure 1, lapatinib was almost as effective as sulforaphane in populations with low percentage of resistant cells, and combined therapy was the most effective. When percentage of lapatinib-resistant cells was more than >25%, lapatinib alone had the lowest efficacy. However, the low viability level was maintained due to sulforaphane activity, either alone or in combination with lapatinib.

### 3.2. Inhibition of Invasion of the Drug-Sensitive and Drug-Resistant SGC-7901 Cells by the Combination of Lapatinib and Sulforaphane

**Figure 2:**
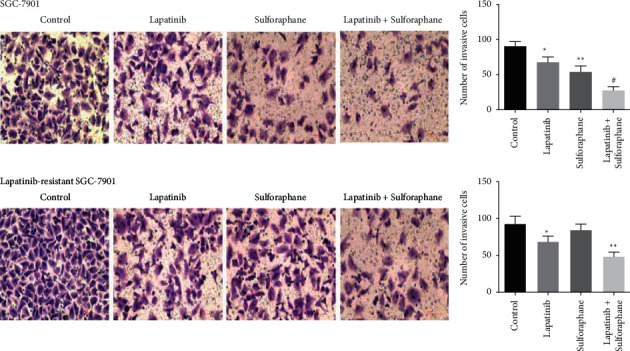
Combination of lapatinib with sulforaphane inhibits cell migration as compared to activity of each agent alone. Data presented the mean value of three independent experiments, ^*∗*^*P* < 0.05, ^*∗∗*^*P* < 0.01, and ^#^*P* < 0.001.

To investigate whether the combined treatment affects cell invasion, we conducted Boyden chamber assay. In Figure 2, cells were treated with lapatinib, sulforaphane, or their combination, the migrations of SGC-7901 and lapatinib-resistant SGC-7901 cells were significantly inhibited compared with control group (*P* < 0.05). In lapatinib-resistant SGC-7901 cell, although sulforaphane alone had no significant effect on cell invasion, combination of lapatinib with sulforaphane was observed to significantly inhibit cell invasion. Moreover, combined treatment most efficiently decreased invasion of SGC-7901 (*P* < 0.001) and lapatinib-resistant SGC-7901 (*P* < 0.001) cells.

### 3.3. The Roles of Lapatinib-Sulforaphane Treatment in Cell Cycle Progression and Cell Apoptosis

**Figure 3:**
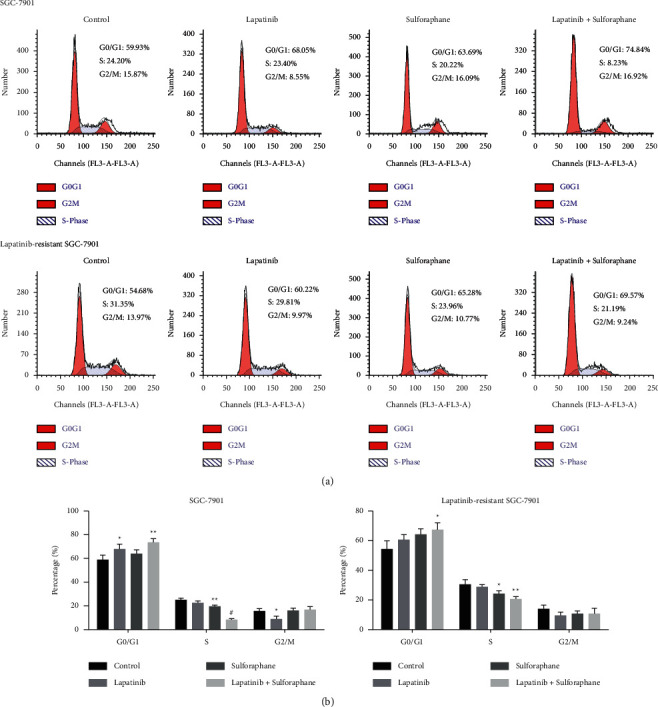
Cell cycle was measured by PI staining assay. (a) Cell cycle of SGC-7901. (b) Cell cycle of lapatinib-resistant SGC-7901. Data presented the mean ± standard deviation of three independent experiments, ^*∗*^*P* < 0.05 and ^*∗∗*^*P* < 0.01.

**Figure 4:**
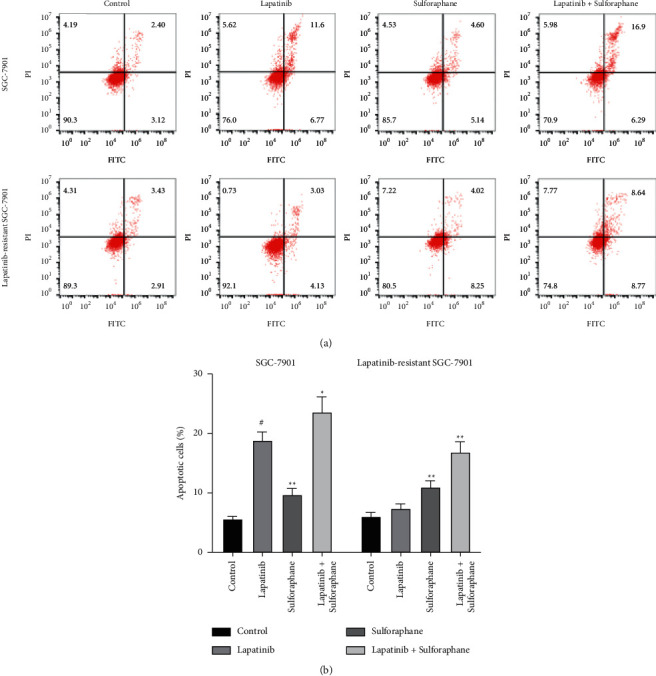
Cell apoptosis was measured by Annexin V-FITC/PI double staining assay. (a) Cell apoptosis of SGC-7901. (b) Cell apoptosis of lapatinib-resistant SGC-7901. Data presented the mean ± standard deviation of three independent experiments, ^*∗*^*P* < 0.05, ^*∗∗*^*P* < 0.01, and ^#^*P* < 0.001.

We explored the effects of lapatinib, sulforaphane, or their combination on the cell cycle of SGC-7901 and lapatinib-resistant SGC-7901 cells. As shown in Figures 3(a) and 3(b), lapatinib, sulforaphane, and combined treatment resulted in an accumulation of cells in the G0/G1 phase and a reduction in the S phase of both cells. Lapatinib significantly induces G2/M arrest in SGC-7901 cell (*P* < 0.05), while not available in lapatinib-resistant SGC-7901. In Figures 4(a) and 4(b), lapatinib-sulforaphane treatment significantly promoted apoptosis of SGC-7901 and lapatinib-resistant SGC-7901 (*P* < 0.05). Sulforaphane is obviously helpful for the effect of lapatinib on cell apoptosis, especially in lapatinib-resistant SGC-7901 (*P* < 0.01).

### 3.4. Lapatinib-Sulforaphane Treatment Downregulated the Expressions of HER-2, p-HER-2, AKT, p-AKT, ERK, and p-ERK

**Figure 5:**
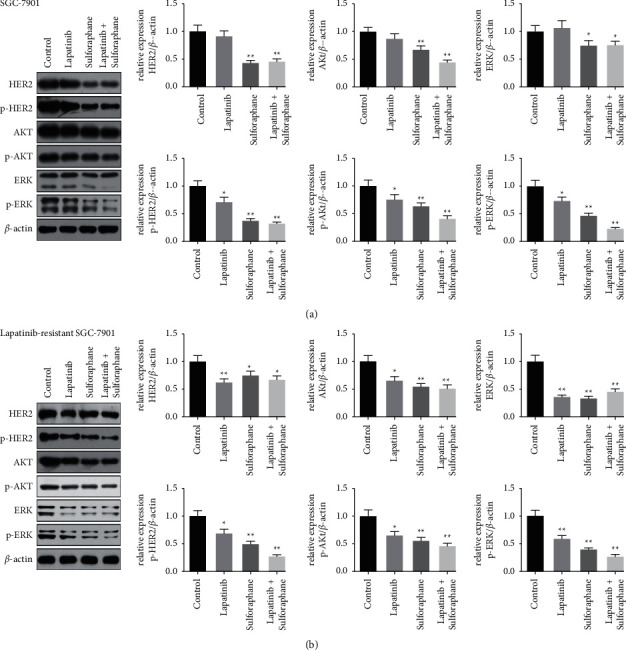
The protein expression levels of HER2, p-HER2, AKT, p-AKT, ERK, and p-ERK were measured by Western blotting. (a) The expressions of HER2, p-HER2, AKT, p-AKT, ERK, and p-ERK in SGC-7901. (b) The expressions of HER2, p-HER2, AKT, p-AKT, ERK, and p-ERK in lapatinib-resistant SGC-7901. Data presented the mean ± standard deviation of three independent experiments, ^*∗*^*P* < 0.05 and ^*∗∗*^*P* < 0.01.

To investigate the possible mechanism underlying the lapatinib-sulforaphane treatment, we detected the expression of genes related to HER2 signaling pathway by Western blotting (Figures 5(a) and 5(b)). In SGC-7901 cells, we observed that lapatinib significantly inhibited the expressions of p-HER-2, p-AKT, and p-ERK (*P* < 0.05), but no remarkable effects on the expressions of HER-2, AKT, and ERK. In addition, HER-2, p-HER-2, AKT, p-AKT, ERK, and p-ERK expressions were significantly lower in sulforaphane and lapatinib-sulforaphane groups than those in the controls (*P* < 0.05). As shown in Figure 5(b), the expressions of HER-2, p-HER-2, AKT, p-AKT, ERK, and p-ERK were significantly downregulated in populations enriched in lapatinib-resistant SGC-7901 cells. In SGC-7901 and lapatinib-resistant SGC-7901 cells, the inhibiting effect of lapatinib-sulforaphane treatment on the expressions of p-HER-2, p-AKT, and p-ERK were more significant than drug alone.

## 4. Discussion

In a large cohort of patients with GC, HER2 overexpression was reported as 11.8% of GC and was significantly related to aging, intestinal histology, clinical stages, venous invasion, and frequent lymph node metastasis [19]. Lapatinib is small molecule inhibitors of HER2, approved by the United States Food and Drug Administration for the treatment of HER2-positive BC patients. Lapatinib also has clinical activity in women with advanced HER2-positive GC [20]. However, primary or acquired resistance against treatment with HER2 inhibitor is an unresolved issue in clinical oncology. Several molecular mechanisms underlying the resistance to lapatinib have been proposed, including mutations within genes of HER2 or EGFR receptors, abnormal expression of hormones, hyperactivation of PIK3Ca, or mutations in PI3K pathway gene [11, 21]. Chen et al. indicated that activation of receptor tyrosine kinases promotes resistance to lapatinib in patients with HER2-positive GC [20].

The combination of phytochemicals with lapatinib is the potential way to overcome drug resistance. Phytochemicals, including sulforaphane, are important tools of cancer prevention and therapy [22]. The anticancer efficacy of sulforaphane was reported to be associated with ROS production, mitosis-specific arrest during cell cycle, and blocking of several signaling pathway [23, 24]. To address the question of whether lapatinib-sulforaphane treatment might be effective for GC and especially lapatinib-resistant GC, we firstly cultured cell-based model of lapatinib resistance using SGC-7901 cells. We further mixed SGC-7901 and lapatinib-resistant SGC-7901 cells in various proportions, which might mimic heterogeneity of gastric tumor in context of the resistance acquisition process. MTT assay revealed lapatinib and sulforaphane synergistically led to a decline in cell viability, which was better than the effect of each agent alone. It is consistent with the efficiency of lapatinib combined with one of isothiocyanates (sulforaphane, erucin, or sulforaphene) in BC, suggesting sulforaphane sensitize lapatinib-resistant cells to the drug [25]. Meanwhile, Angelika observed that combination of lapatinib with sulforaphane caused a decline in cell invasion [25]. Our study confirmed it and showed lapatinib-sulforaphane was effective in inhibiting invasion of SGC-7901 and lapatinib-resistant SGC-7901 cells.

In cancer cells, inhibition of cell cycle regulation is a potential cancer treatment. Previous studies indicated that sulforaphane suppressed the proliferation of cancer cells by inducing G1 or G2/M arrest in multiple cancer cells [26]. YUNG reported sulforaphane inhibited proliferation of AGS human GC cells by inducing the cellular portion of the G2/M phase, whereas lapatinib was only caused in a significant G1 arrest in lapatinib-sensitive BC cell [7]. In the present study, lapatinib significantly induced G0/G1 arrest in SGC-7901 cell and sulforaphane led to a decrease of S phase in SGC-7901 and lapatinib-resistant SGC-7901 cell. The combination of lapatinib and sulforaphane resulted in an accumulation of cells in the G0/G1 phase and a reduction in the S phase of both cells. This is the first time to confirm that lapatinib-sulforaphane regulated cell cycle in SGC-7901 and lapatinib-resistant SGC-7901 cells.

Apoptosis is a physiological process that regulates cell death. Inducing apoptosis is one of the common methods to control cancer progression. Lapatinib inhibited the growth of HER2 gene-amplified trastuzumab-sensitive and resistant GC cells by inducing apoptosis [27], whereas lapatinib had no impacts on lapatinib-resistant BC cells [7]. Meanwhile, sulforaphane was reported to induce apoptosis of cancer cells through AMPK-dependent or SHH pathway [28, 29]. Our results were similar to previous researches. We found lapatinib induced apoptosis in SGC-7901 cell, and sulforaphane had significantly impact on both cells. The combination of lapatinib and sulforaphane was the most effective agent to inducing apoptosis of SGC-7901 and lapatinib-resistant SGC-7901 cells. It indicates that sulforaphane could enhance the effects of lapatinib on apoptosis in drug-sensitive and drug resistant cells.

To investigate the functional mechanism of lapatinib combined with sulforaphane on GC cells, the expressions of HER2 signaling pathway related genes were measured. Akt and ERK were the main HER2 downstream effectors, which played an important role inPI3/AKT and MAPK signaling pathway to regulate the growth, apoptosis, and death of cells. In this study, lapatinib significantly downregulated the expressions of p-HER2, p-AKT, and p-ERK, but lapatinib reduced the expressions of HER2, AKT, and ERK in lapatinib-resistant cells. It revealed that the drug resistance of SGC-7901 cell may be related to HER2, AKT, and ERK proteins expression. On the other hand, lapatinib-sulforaphane treatment significantly inhibited PI3/AKT and MAPK signaling pathway, especially in the decreased expressions of p-HER2, p-AKT, and p-ERK. The similar results were also shown in BC and HER2-positive GC [4, 25].

In conclusion, we investigated the molecular mechanism of lapatinib combined with sulforaphane in SGC-7901 and lapatinib-resistant SGC-7901 cells. Our results suggested that lapatinib-sulforaphane might be a novel and promising agent in both lapatinib-sensitive and lapatinib-resistant GC patients.

## Data Availability

All data are available within the article.
